# Self-perceived attitudes of Italian oncology nurses towards clinical trial involvement: A cohort observational study

**DOI:** 10.1177/03008916241290736

**Published:** 2024-10-27

**Authors:** Elsa Vitale, Roberto Lupo, Luana Conte, Rocco Mea, Ivan Rubbi, Serena Iacovelli, Giorgio De Nunzio, Raffaella Massafra

**Affiliations:** 1 Scientific Directorate, IRCCS Istituto Tumori Giovanni Paolo II, Bari, Italy; 2 San Giuseppe da Copertino Hospital, Local Health Authority of Lecce, Copertino, Lecce, Italy; 3 Laboratory of Biomedical Physics and Environment, Department of Mathematics and Physics E. De Giorgi, University of Salento, Lecce, Italy; 4 Laboratory of Advanced Data Analysis for Medicine (ADAM) at the Laboratory of Interdisciplinary Research Applied to Medicine, University of Salento and Local Health Authority of Lecce, Lecce, Italy; 5 Cardiology Unit, San Carlo Hospital, Potenza, Italy; 6 School of Nursing, University of Bologna, Faenza, Italy; 7 Laboratorio di Bioinformatica e Biostatistica, IRCCS Istituto Tumori Giovanni Paolo II, Bari, Italy

**Keywords:** Attitude, clinical trial, oncology nursing

## Abstract

**Background:**

Literature is lacking when it comes to oncology nursing attitudes in clinical trial involvement.

**Objective:**

To assess how Italian oncology nurses perceived their attitudes in clinical trials involvement.

**Methods:**

An on-line cohort observational study was carried out. Data collected included: sex, years of work experience in oncology field and 10 items assessing participants’ self-perceptions of their attitudes in clinical trials. Linear regression was used to assess associations between work experience and self-perceived preparedness.

**Results:**

A total of 338 Italian oncology nurses were enrolled. Most nurses declared not receiving any specific training in oncology clinical trials (23.1%). No significant associations were reported between self- perceived attitudes in clinical trial involvement in the oncology setting and both work experience and clinical trial involvement.

**Conclusions:**

Cancer centers are improving cancer nursing research in supplying clinical care. But very few centers are involved in training oncology nurses. This gap seems to be very deep in taking into consideration the oncology nursing research in clinical trials, too.

## Introduction

The World Health Organization (WHO) drew attention to the worldwide lack of nurses in the publication of State of the World’s Nursing Report in 2020, which states that by 2030 the deficiency would only improve, especially in low and lower-middle income countries.^1^ This condition has a direct impact on the number of nurses employed in care cancer with very few nurses actually available to population.^1,2^ In this regard, the WHO introduces policies for nursing training, work tasks, leadership, and service supply in order to improve ability to handle the public health impacts from the development of the cancer burden from a nursing point of view, since oncology nurses should be involved globally both in cancer advance tracking and its related prevention.^3^ They are also included in several dimensions of cancer treatment, such as: complementary medicine,^4^ shared decision-making,^5^ end-of-life^6^ and survivorship.^7^ Oncology nurses should also take part in oncology research, especially in supportive^6^ and psychosocial care support^8^ in order to guarantee care and better patient and family quality of life. Globally, standard training in oncology includes a graduate program or a board certification supported by a curriculum in line with the European Society for Medical Oncology and ASCO recommendations.^9,10^ Evidence suggests that multidisciplinary partnerships covering several dimensions, like chemotherapy and radiation administration,^11,12^ nursing care^13^ and cancer screening^14^ seems to be essential for improving cancer goals.

Thus, oncology nursing needs specialized and clinical training in order to supply cancer nursing security along with the cancer continuum process. This must be achieved through global nursing training and experience, in order to reach competency standards, continuing training and oncology nursing leadership across cancer control and planning activities. In current nursing there is a lack of specialized oncology nursing schools, too. However, the strong increase in cancer epidemiology everywhere, draws attention to the oncology nursing skills urgently required to support this specific patient population. In this regard, international authorities suggest healthcare systems quantify their oncology nursing workforce worldwide in order to improve general population survival rates.^15^ In Europe, the increasing in specialized oncology nursing education is not standardized between the western and eastern countries.^16^ In fact, most of specialized educational programs vary in each European country, as some provide a university-level master’s degree and others a university-based or specialized cancer hospital training program.^16^ On the other hand, in America, oncology nursing training programs take place in hospitals with formal oncology nursing orientation programs for new nurses, with a preceptorship through a senior oncology nurse for up to 1 year.^17^ In the USA, oncology nursing programs do not supply nurses with a postgraduate coursework or a master’s degree in oncology. However, there is a lack of literature regarding oncology nursing attitudes in clinical trial involvement. In this scenario, the present study aimed to assess how Italian oncology nurses perceived their attitudes in clinical trial involvement. To reach these findings and investigate the potential areas that are lacking for training in cancer research and clinical practice in Italy, we promoted a questionnaire to spread among Italian oncology nurses exploring self-perceptions in training in oncology practice and research for oncology nurses in Italy. Specifically, the present study explored self-perceptions in preparedness and any variations also according to years of work experience in oncology facilities and the nursing involvement in clinical trials, respectively.

## Materials and methods

### Study design

A cohort observational study was carried out during February 2024.

### Participants

All Italian oncology nurses employed in different oncology settings, both hospital and territory, could potentially be included, with the exclusion of retired nurses.

### Questionnaire administration

An on-line questionnaire was created and spread through the web site ‘Nurseallface’ social page, inviting nurses employed in the oncology field to complete the questionnaire. All visitors could access the presentation letter stating the intent of the proposed study and only those who gave consent to participate and declared to be an oncology nurse could proceed further into the questionnaire.

### Data collection

Sampling characteristics included: sex (female and male), years of work experience in oncology field (less than 5 years, 6-10 years, 11-15 years, 16-20 years, 21-25 years, 26-30 years, over 31 years). Finally, a total of 10 items were proposed to assess participants’ self-perceptions in their attitudes in clinical trial involvement in the oncology setting. The questionnaire contained a total of 9 questions in which each participant self-assessed their perceptions in their own expertise, preparation, willingness, training level in research methodology or in participation, preparedness as principal or sub principal investigator (PI). For each item a Likert scale was associated varying from 1, as “insufficient” to 5, as “excellent”. Only the fifth item investigated what type of training the participant received in oncology clinical trials among those proposed, such as: no teaching, conferences or congresses, small group learning, online teaching modules, independent learning, training delivered by the clinical trial department, learning from clinical practice, accredited training programs or others.

### Validation of the items proposed

Items were created ad-hoc taking into consideration the current literature available.^18,19^ Then, 10 oncology nurses were queried to see if the questionnaire covered all the aspects of the basic clinical trial skills in the oncology nursing field and the “Survey Instrument Validation Rating scale” was proposed to assess how the questions could correspond with the real world investigated.^20^ The validation checklist included a total of 13 criteria in which participant should give a degree of agreement or disagreement corresponding to the best judgement related to the reality of their experiences. A Likert scale was associated ranging from 1 as “strongly disagree” to 5 as “strongly agree”. Criteria investigated included the relevance of the objectives of the study, the depth of the instrument to constructs being assessed, the limits stated, the clarity, if the responses were stated in a clear manner and appropriate to the context proposed, the layout or format of the instrument, the range of answer proposed, the length and the interest of the questionnaire which should reflect the reality experienced by responses.

All nurses answered as “strongly agree” in their validation assessment. Additionally, the items proposed recorded a good validity as α=0.812.

### Study size

In 2021, the Italian Ministry of Health, discovered that about 59.2% from the total of Italian healthcare professionals (n=617,246) were nurses.^21^

By considering the Miller and Brewer’s formula^22^ and fixing the confidence interval at 95%, sample size was assessed by considering the formula: n= N/(1+N(α)^2^), where n indicated the desired sample size, N the target population and α the level of statistical significance of 0.05 and 1 was a constant. Therefore, the sample size assessment was: n = 365,410 / (1+(365,410 (0.05)^2^) = 400, for all the nursing specializations. However, there was very little evidence indicating the specific numbers for each nursing specialty. The Italian Healthcare System contains nearly 70 clinical specialties,^23^ therefore, we could consider a simple size of 300 in nursing oncology specialization as representative of the Italian context.

### Statistical methods

Data were collected in an Excel datasheet and then, statistics were performed. Sex, work experience in oncology field, training performed in clinical trials declared and all the items proposed were considered as categorical variables and presented as frequencies and percentages. Age was expressed in years and showed as mean ± standard deviation. Then, linear regressions were performed between years of work experience in oncology setting and the nursing involvement in clinical trials in each item of nursing self-perceptions. The significant level was assessed at p < 0.05. Finally, frequencies and percentages were assessed for all significant associations registered in order to assess the various associations highlighted.

### Ethical considerations

In the first part of the questionnaire a clear presentation of the study and its related purpose was described to inform all the potential participants. According to the Committee on Publication Ethics (COPE),^24^ the questionnaire was anonymous and anonymity was maintained during the research and publication processes. It was stated that the participation was voluntary and participants who wished to participate should give their individual informed consent. Additionally, in 2020, the Italian Superior Institute of Health introduced all the functions owed the Italian Ethical Committee (EC). Therefore, the EC should express opinions on protocols of clinical drug trials, observational clinical trials, clinical trials with medical devices, or protocols for therapeutic use of investigational drugs outside clinical trials or for biomedical, psycho-educational, social or other research involving human subjects humans; epidemiological, evaluative and medico-social research projects that require the collection of data personal data or with environmental ethics implications; patient information sheets and informed consent forms; ethical-scientific, methodological and economic aspects of experimental research protocols or amendments; qualification of investigators for the purpose of conducting the proposed research as well as the ethical and scientific aspects of the same. As the present study investigated how Italian oncology nurses self-perceived their attitudes in the clinical trial involvement by also considering their years of work experience in oncology stings and their past involvements in clinical trials, the EC opinion was omitted in its request.

## Results

A total of 338 Italian oncology nurses were enrolled in this study. Of these, 63.3% were female and 36.7% were male and all were aged 36±9 years (Table 1). Most of participants (49.1%) stated they had less than five years of work experience in oncology field. Finally, most of the nurses stated they did not receive any specific training in oncology clinical trials (23.1%) or received specific training for this specific issue from another, non-institutionalized, form of training.

**Table 1.** Sampling characteristics of oncology Italian nurses (n=338).

Sampling characteristics	n(%); µ±s.d.
Sex	
Female	214(63.3%)
Male	124(36.7%)
Age	36±9
Work experience in Oncology settings	
Up to 5 years	166(49.1)
6-10 years	66(19.5)
11-15 years	35(10.4)
16-20 years	24(7.1)
21-25 years	24(7.1)
26-30 years	7(2.1)
More than 31 years	16(4.7)
Training performed in clinical trials	
Learning from clinical practice	81(24)
Learning in small groups	10(3)
Independent learning	15(4.4)
Training delivered by the clinical trials department	18(5.3)
Online teaching modules	28(8.3)
No teaching	78(23.1)
Accredited training programs	36(10.7)
Other	72(21.3)

*Abbreviations*: n: frequency; µ: mean; s.d.: standard deviation.

**Figure 1.**
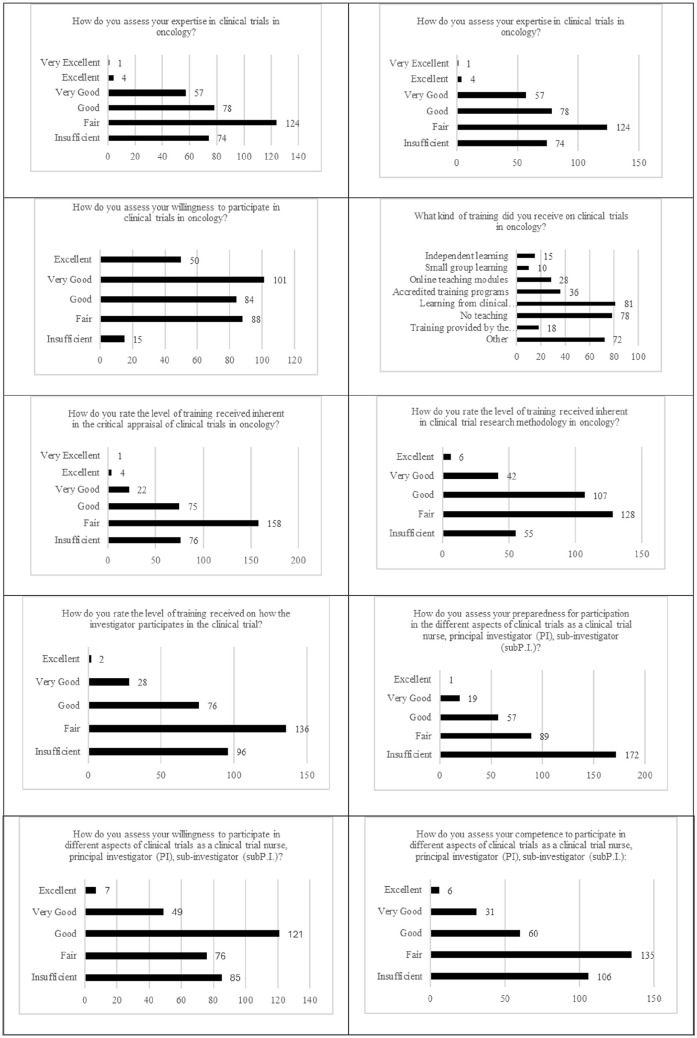
Self-perceptions on oncology nursing training in clinical trials (n=338).

By considering the items proposed in self-perceptions of oncology nursing training in clinical trials, as shown in Table 2, most of nurses declared to have a poor self-perception in their attitudes in all the dimensions investigated, such as own expertise, since only 1.2% of nurses defined it as excellent. As regards preparation, none among the interviewed nurses quantified this attitude as excellent, although 14.8% of nurses declared having excellent willingness to take part in clinical trials. However, only 1.2% of participants defined the training received in this issue as excellent and 1.8% the related methodology learned, that decreased as 0.6% as excellent self-perception in training received to be part in a clinical trial as principal investigator (P.I.) and 0.3% to be part as a sub P.I. with 1.8% of nurses who self-assessed their attitudes as excellent to be part in a clinical trial both as P.I. or sub P.I (Figure 1).

**Table 2.** Linear regression between self-perceived preparedness and work experience in oncology setting.

Self-perceived preparedness Items proposed	Self-perceived preparedness			
	β	t	C.I. 95% Min-Max	p-value
Item no.1	0.064	0.799	−0.285–0.463	0.425
Item no.2	−0.039	−0.484	−0.151–0.364	0.629
Item no.3	−0.078	−1.170	−0.389–0.235	0.243
Item no.4	−0.080	−1.160	−0.324–0.079	0.270
Item no.5	0.072	1.079	−0.433–0.117	0.282
Item no.6	−0.143	−1.864	−0.110–0.361	0.063
Item no.7	−0.005	0.106	−0.535–0.020	0.916
Item no.8	0.125	−0.072	−0.246–0.279	0.943
Item no.9	0.125	1.512	−0.243–0.231	0.131

* p<0.05 is statistically significant.

Furthermore, associations were assessed between self-perceived attitudes in clinical trial involvement in the oncology setting and work experience (Table 2). However, no significant associations were reported, so older oncology nurses seemed not to have higher attitudes in oncology clinical trials than the younger ones, respectively.

Finally, associations were assessed between self-perceived attitudes in clinical trials in the oncology setting and involvement in clinical trials (Table 3). No significant associations were reported also in this relationship, so the active involvement in clinical trials seemed not to represent an incentive in learning more about this topic.

**Table 3.** Linear regression between self-perceived preparedness and the oncology nursing involvement in clinical trials.

Self-perceived preparedness Items proposed	Self-perceived preparedness			
	β	t	C.I. 95% Min-Max	p-value
Item no.1	−0.046	−0.579	−0.097–0.053	0.563
Item no.2	0.000	−0.001	−0.091–0.091	0.999
Item no.3	0.069	1.020	−0.028–0.089	0.309
Item no.4	0.070	0.960	−0.041–0.119	0.338
Item no.5	0.076	1.126	−0.029–0.108	0.261
Item no.6	−0.089	−1.151	−0.128–0.033	0.251
Item no.7	−0.043	−0.587	−0.099–0.054	0.558
Item no.8	−0.072	−0.951	−0.102–0.036	0.343
Item no.9	−0.011	−0.127	−0.087–0.076	0.899

* p<0.05 is statistically significant.

## Discussion

The present study aimed to assess how Italian oncology nurses perceived their attitudes in clinical trial involvement. Specifically, if the perceived preparedness changed according to both years of work experience in oncology setting and the nursing involvement in clinical trials, respectively.

We know that every day, oncology nurses deliver compound care in stress-evoking conditions, embracing end-of-life care, supplying bad news and treatments to patients suffering from both the disease and treatment side-effects, with families who are just overpowered by their family member’s suffering.^25^ Oncology nurses usually experience ethical difficulties related to cancer care^26,27^ and relate to their patients who may lose health or life.^28^ Additionally, oncology nursing has a wide aim, since it deals with screening, genetic counseling, nurse navigation, advanced practice nurses with a very limited publicly acknowledged success. In this scenario the importance of nurses in oncology research and in clinical trial conduction, should also be added. It may imply that oncology nursing involves great levels of patient-to-nurse relationships translating into heavy workloads, which contribute to job stress and burnout.^29^ In the present study, findings highlighted that most of the nurses did not receive adequate specific training for oncology clinical trials (23.1%) or received it from other, non-institutionalized form of training for this specific issue. The findings established that more than half of the respondents had no standard oncology training in either pre or post professional oncology education. These findings overlap with the scant evidence available in the current literature in which oncology nurses received their cancer-related education through workshops, seminars, and online/virtual trainings, mostly self-funded.^30^ The condition gets worse if we considered oncology clinical trials, since in the items proposed in self-perceptions in oncology nursing training in clinical trials, most of nurses declared to have a poor self-perception in their attitudes in all the dimensions investigated, such as own expertise, since only 1.2% of nurses defined it as excellent. As regards preparation self-perception 0% of nurses quantified this attitude as excellent, although 14.8% of nurses declared to have excellent willingness to take part in clinical trials. However, only 1.2% of participants defined the training received in this issue as excellent and 1.8% the related methodology learned, that decreased as 0.6% as excellent self-perception in training received to be part in a clinical trial as principal investigator (P.I.) and 0.3% to be part as a sub P.I. with 1.8% of nurses who self-assessed their attitudes as excellent to be part in a clinical trial both as P.I. or sub P.I. These findings were in agreement with the study conducted in Nigeria,^31^ in which most of participants assessed their oncology training condition as lower than the standard requested in studies that belonged to different regions all around the world.^30,32^ Similarly, in Turkey, more than half of the nurses in one study did not attend any educational courses in oncology palliative care.^33^ Furthermore, no significant associations were reported, so older oncology nurses seemed not to have higher attitudes in oncology clinical trials than the younger ones, respectively.

Additionally, no significant associations were reported in this relationship also, so that active involvement in clinical trials seemed not to represent an incentive for learning more about this topic.

Also in these aspects, our results were in agreement with the current literature, since the majority of participants in our study did not have specialized trainings in cancer research and statistical analysis, similar to the majority of participants in the Adejumo et al. study^29^ which recorded the lack of ability to carry out oncology research. In this specific field, mentoring and training were also recognized as valuable methods to develop research attitudes,^31^ also including communication and emotional regulation skills highlighted in further studies.^34,35^

Similar findings, although in oncology care supply, were reported in Kenya, where oncology nurses suggested lack knowledge regarding cervical cancer patients.^32,36^ Additionally, in the United Kingdom, the inability to understand and handle symptoms and their related seriousness and highlighted among symptoms of cancers.^36,37–39^ However, no research study provided specific training in oncology nursing and outcome assessments, too.

However, the present study had some limitations. First, data were collected in an online mode, and this approach may have partially excluded those who have lower technological ability. Moreover, much information bias may be due to a hostile behavior in declaring and, therefore, admitting the effective condition explored. Finally, in the questionnaire administered, we did not include and discuss potential reasons why nurses have poor self-perception regarding clinical trials, such as the quality of available training, workplace support, and cultural attitudes towards clinical research, also including qualitative data, such as interviews or open-ended survey responses, which could provide richer insights into the nurses’ attitudes and experiences.

## Conclusion

Cancer centers are improving cancer nursing research in supplying clinical care. But very few centers are involved in training oncology nurses. This gap seems to be very wide when taking into consideration the oncology nursing research in clinical trials, too. Therefore, significantly more could be done with increased attention paid to this often-invisible part of oncology care delivery. Providing nurses with improved access to information, training, and recognition of specialization could be a major advance in cancer care in several oncology nursing settings, which serve extremely large numbers of cancer patients.^28,40^
